# Integrated Multi-Omics Links Bisphenol AF (BPAF) Exposure to Hepatic Lipid Metabolism Disruption via Succinate Dehydrogenase Dysfunction and Mitochondrial Impairment

**DOI:** 10.3390/metabo16070440

**Published:** 2026-06-24

**Authors:** Ning Wang, Jing Xu, Jing Leng, Jia-Le Xu, Da-Sheng Lu, Fan Zhang, Dong-Sheng Yu, Ke-Lei Qian, Gong-Hua Tao, Ping Xiao, Xin-Yu Hong

**Affiliations:** Shanghai Municipal Center for Disease Control and Prevention, Institute of Chemical Safety Evaluation/Key Laboratory of Environmental and Health Impact Assessment of New Pollutants, Shanghai 201107, China; xujing@scdc.sh.cn (J.X.); lengjing@scdc.sh.cn (J.L.); xujiale@scdc.sh.cn (J.-L.X.); ludasheng@scdc.sh.cn (D.-S.L.); zhangfan@scdc.sh.cn (F.Z.); yudongsheng@scdc.sh.cn (D.-S.Y.); qiankelei@scdc.sh.cn (K.-L.Q.); taogonghua@scdc.sh.cn (G.-H.T.)

**Keywords:** bisphenol AF (BPAF), hepatotoxicity, multi-omics, succinate dehydrogenase, tricarboxylic acid (TCA) cycle, lipid metabolism, mitochondrial dysfunction

## Abstract

**Background/Objective:** Bisphenol AF (BPAF), a fluorinated analogue of bisphenol A, is an environmental contaminant associated with hepatotoxicity and metabolic disruption. However, the systematic molecular mechanisms linking early transcriptional events to metabolic dysfunction in the liver remain poorly defined. The aim of this study is to elucidate the association between BPAF exposure and hepatic lipid accumulation by integrating transcriptomics, cellular metabolomics, and targeted phenotypic assays. **Methods:** We performed RNA-sequencing on livers from mice exposed to BPAF (0.1–10 mg/kg/day, 28 days), and performed non-targeted metabolomics on AML12 murine hepatocytes co-cultured with RAW264.7 macrophages in a Transwell system (0–2500 nM BPAF, 48 h). Key metabolic pathways were identified through integrated bioinformatics and validated using enzymatic assays, qRT-PCR, Western blotting, and phenotypic staining (lipid droplets, ROS). **Results:** Multi-omics integration revealed significant disruption of PPAR signaling and the tricarboxylic acid (TCA) cycle. A striking dose-dependent accumulation of succinate was observed in exposed cells, concomitant with a significant inhibition of succinate dehydrogenase (SDH) activity (52% reduction at 2500 nM, *p* < 0.001). Transcriptomic data confirmed the downregulation of mitochondrial fatty acid β-oxidation genes. Phenotypic validation indicated that BPAF exposure is associated with oxidative stress, pro-inflammatory cytokine release (TNF-α, IL-6), and pronounced intracellular lipid droplet accumulation in hepatocytes. **Conclusions:** This study suggests that BPAF exposure is associated with SDH dysfunction, TCA cycle arrest, and lipid dysregulation. Whether BPAF directly inhibits SDH or acts through upstream mitochondrial targets warrants further structural and kinetic investigation.

## 1. Introduction

Bisphenol AF (BPAF) is a fluorinated analogue of bisphenol A (BPA) widely utilized in the manufacturing of polycarbonate copolymers, fluoroelastomers, and electronic materials [1]. Due to its widespread use and environmental persistence, BPAF is frequently detected in surface water, sediment, and indoor dust, leading to chronic human exposure primarily through dietary intake [2]. Increasing toxicological evidence indicates that BPAF exerts greater endocrine-disrupting potential and metabolic toxicity than its parent compound BPA [3,4,5].

While the endocrine effects of BPAF have been extensively characterized, emerging evidence points to the liver as a primary target organ for BPAF-induced metabolic disruption. BPAF exhibits estrogenic, anti-androgenic, and thyroid-disrupting activities, which have been linked to reproductive, developmental, and metabolic abnormalities in animal models. Studies in animal models and in vitro systems have reported that BPAF exposure promotes hepatic steatosis, alters lipid profiles, and induces oxidative stress [6]. However, the precise molecular cascade—specifically how BPAF exposure transduces signals from initial gene expression changes to functional metabolic failure and lipid accumulation—remains incompletely understood.

Traditional toxicological approaches often rely on single-endpoint assays, which may fail to capture the complexity of cellular responses to xenobiotic stress. To address this gap, we employed an integrated New Approach Methodology (NAM) framework combining transcriptomics and metabolomics. Transcriptomics provides a genome-wide snapshot of cellular adaptive or adverse responses, while metabolomics offers a functional readout of downstream biochemical activity [7].

In this study, we aimed to investigate whether BPAF exposure is associated with disruption of mitochondrial function, with a specific focus on the tricarboxylic acid (TCA) cycle. Previous transcriptomic studies have implicated PPAR and fatty acid oxidation pathways, but the upstream mitochondrial event has remained elusive. Given that several bisphenols are known to impair mitochondrial respiration, we hypothesized that BPAF exposure interferes with TCA cycle function, potentially via succinate dehydrogenase (SDH). To test this hypothesis, we performed RNA-seq on livers from BPAF-exposed mice and non-targeted metabolomics on a murine hepatocyte–macrophage co-culture model [8,9]. Our integrated analysis identified an association between SDH dysfunction, succinate accumulation, and hepatic lipid dysregulation.

## 2. Materials and Methods

### 2.1. Animal Exposure and Transcriptomics

Animal Study: Male C57BL/6 mice (6–8 weeks old) were housed under standard conditions (22 ± 2 °C, 12 h light/dark cycle) with ad libitum access to food and water. All animal procedures were approved by the Animal Ethics Committee of the Shanghai Municipal Center for Disease Control and Prevention (IACUC-PZ-2024-038). Following a one-week acclimation, mice were randomly divided into four groups (*n* = 6 per group) and administered BPAF (purity > 98%, Sigma-Aldrich, St. Louis, MO, USA) dissolved in corn oil via oral gavage at doses of 0, 0.1, 1, or 10 mg/kg body weight per day for 28 consecutive days.

Dose selection rationale: The doses (0.1, 1, and 10 mg/kg/day) were chosen to span from environmentally relevant levels (based on prior literature and human biomonitoring) to a top dose suitable for hazard identification, consistent with OECD guidelines and previous BPAF toxicology studies [6].

Randomization: Mice were randomly assigned to groups using a computer-generated random number sequence (Random.org).

Vehicle control: Corn oil alone (vehicle) was administered to the control group. Preliminary experiments confirmed that corn oil had no significant effect on any measured endpoint compared to naïve controls.

Euthanasia: Mice were euthanized by carbon dioxide inhalation followed by cervical dislocation, in accordance with institutional animal care guidelines.

RNA Sequencing and Data Analysis: Twenty-four hours after the final dose, mice were euthanized, and liver tissues were rapidly excised and stored in RNAlater (Thermo Fisher Scientific, Waltham, MA, USA). Total RNA was extracted using TRIzol reagent (Thermo Fisher Scientific, Waltham, MA, USA). Library preparation and sequencing were performed on a DNBSEQ-T7 platform (MGI Tech, Shenzhen, China) with 150 bp paired-end reads. Raw reads were filtered and aligned to the mouse reference genome (mm10) using STAR aligner (v2.7, Cold Spring Harbor Laboratory, Cold Spring Harbor, NY, USA). Gene expression counts were quantified using HTSeq (v0.11, Python package, https://htseq.readthedocs.io), and differential expression analysis was conducted using DESeq2 (v1.30, Bioconductor; Boston, MA, USA) with a threshold of |log_2_ Fold Change| ≥ 1 and false discovery rate (FDR) < 0.05. Gene Ontology (GO) and Kyoto Encyclopedia of Genes and Genomes (KEGG) pathway enrichment analyses were performed using the clusterProfiler version 4.10.0 (R package, Guangzhou, China). RNA integrity was confirmed with a Bioanalyzer (Agilent Technologies, Santa Clara, CA, USA) (mean RIN > 8.0). Paired-end reads (150 bp) were sequenced to an average depth of ~40 million clean reads per sample, of which >85% uniquely aligned to the mm10 reference genome. Differential expression analysis was performed with DESeq2 using the design formula~dose; no batch correction was necessary as all samples were processed in a single sequencing run.

### 2.2. Metabolomics Analysis of Co-Cultured Cells

A flowchart summarizing the overall study design is provided in Figure S2 (Supplementary Materials).

Cell Culture and Transwell Co-culture System: AML12 murine hepatocytes and RAW264.7 murine macrophages were obtained from the Chinese Academy of Sciences Cell Bank (Shanghai, China). To mimic the liver microenvironment, a non-contact Transwell co-culture system was established. RAW264.7 cells (5 × 10^4^ cells/insert) were seeded in the upper chamber of a 6-well Transwell insert (3.0 µm pore size; Corning, Corning, NY, USA), while AML12 cells (2 × 10^5^ cells/well) were seeded in the lower chamber. Cells were maintained in a 1:1 mixture of RPMI 1640 and DMEM/F12 medium (Gibco, Thermo Fisher Scientific, Waltham, MA, USA) supplemented with 10% fetal bovine serum (FBS, Gibco, Thermo Fisher Scientific, Waltham, MA, USA). After 24 h of attachment, cells were exposed to BPAF at concentrations of 0, 100, 500, or 2500 nM for 48 h (*n* = 6 biological replicates). The concentration range (100–2500 nM) was selected to bracket environmentally relevant to supra-environmental levels based on prior in vitro studies of bisphenols and cytotoxicity testing (CCK-8 assay showed >90% viability at all tested concentrations). The lowest concentration (100 nM) is above typical general population serum levels (~0.22 nM) but provides a safety margin for bioaccumulation and is relevant for high-exposure or occupational scenarios.

Metabolite Extraction and UPLC-MS Analysis: Following exposure, culture media from the upper chamber (macrophage compartment) and lower chamber (hepatocyte compartment) were collected separately. Intracellular metabolites were extracted using ice-cold methanol/chloroform/water (Sinopharm Chemical Reagent Co., Shanghai, China) (8:1:1, *v*/*v*/*v*) after washing cells with cold ammonium acetate. Samples were sonicated and centrifuged, and supernatants were dried under nitrogen. Non-targeted metabolomics was performed using an ACQUITY UPLC I-Class system coupled to a Q Exactive Orbitrap mass spectrometer (Thermo Fisher Scientific, Waltham, MA, USA) with a BEH HILIC column (Waters Corporation, Milford, MA, USA). Metabolites were identified using Progenesis QI (Waters Corporation, Milford, MA, USA) and matched against the Human Metabolome Database (HMDB). Differential metabolites were defined by |fold change| ≥ 1.5, VIP > 1, and *p* < 0.05. Quality control (QC) procedures, data filtering criteria and normalization methods are detailed in Supplementary Methods S1.3. Briefly, a pooled QC sample was injected every ten samples; features with a relative standard deviation (RSD) > 30% in QC samples or detected in < 80% of samples were removed. Data were normalised by total ion intensity and log_2_-transformed.

### 2.3. In Vitro Phenotypic Validation

Succinate Dehydrogenase (SDH) Activity: SDH activity in RAW264.7 cell lysates was measured using a commercial assay kit (Sigma-Aldrich, St. Louis, MO, USA) according to the manufacturer’s instructions. Absorbance was measured at 600 nm.

Lipid Droplet Detection: AML12 cells in the lower chamber were fixed with 4% paraformaldehyde and stained with 0.1% Nile Red solution (Beyotime Biotechnology, Shanghai, China) for 15 min at room temperature in the dark. After washing with PBS, fluorescence was visualized using a Leica DM3000 fluorescence microscope (Leica Microsystems, Wetzlar, Germany).

Oxidative Stress and Inflammation: Intracellular reactive oxygen species (ROS) levels were assessed using the DCFH-DA fluorescent probe (Beyotime Biotechnology, Shanghai, China). Levels of tumor necrosis factor-alpha (TNF-α), interleukin-6 (IL-6), and interleukin-1 beta (IL-1β) in cell culture supernatants were quantified using ELISA kits (Beyotime Biotechnology, Shanghai, China).

Quantitative Real-Time PCR and Western Blotting: Total RNA was extracted from AML12 cells, reverse-transcribed, and subjected to qRT-PCR using specific primers (Table S1). For protein analysis, cells were lysed, and proteins were separated by SDS-PAGE, transferred to PVDF membranes (MilliporeSigma, Burlington, MA, USA), and probed with primary antibodies against Akt1, Gsk3b, JNK1/MAPK8, and NF-κB p65 (Beyotime Biotechnology, Shanghai, China) (detailed antibody information provided in Table S2).

### 2.4. Statistical Analysis

Data are presented as mean ± standard deviation (SD). For the in vivo study, *n* = 6 represents individual animals per group; each animal was treated as an independent biological replicate. In the RNA-seq analysis, each animal was processed as a separate sample. For in vitro experiments, metabolomics was performed on six independent biological replicates from separate cell passages and treatments. qRT-PCR was performed in duplicate wells per biological replicate, and Western blotting results shown are representative of three independent experiments. Cytotoxicity (CCK-8) and ELISA assays were conducted in three independent experiments, each with triplicate wells, and the independent experiment was used as the statistical unit.

Prior to parametric testing, data normality was assessed by the Shapiro–Wilk test and homogeneity of variances by Levene’s test. When assumptions were violated, the Kruskal–Wallis test with Dunn’s post hoc test was used, or data were log-transformed. Statistical significance between groups was otherwise determined by one-way ANOVA followed by Dunnett’s post hoc test or Student’s *t*-test where appropriate. A *p*-value < 0.05 was considered statistically significant. Benchmark dose (BMD) modeling was performed using PROAST software (version 65.6, RIVM, Bilthoven, The Netherlands) with model averaging across exponential (3,4,5), Hill (3,4,5), linear, quadratic, and power models. The best-fitting model was selected based on Akaike Information Criterion (AIC), visual inspection of fit, and a BMDL/BMD ratio < 10. The benchmark response was set at a 10% change from control (BMDL_10_). Confidence intervals were calculated using the profile likelihood method.

## 3. Results

### 3.1. Transcriptomic Analysis Reveals Downregulation of Mitochondrial Metabolic Pathways

**Figure 1:**
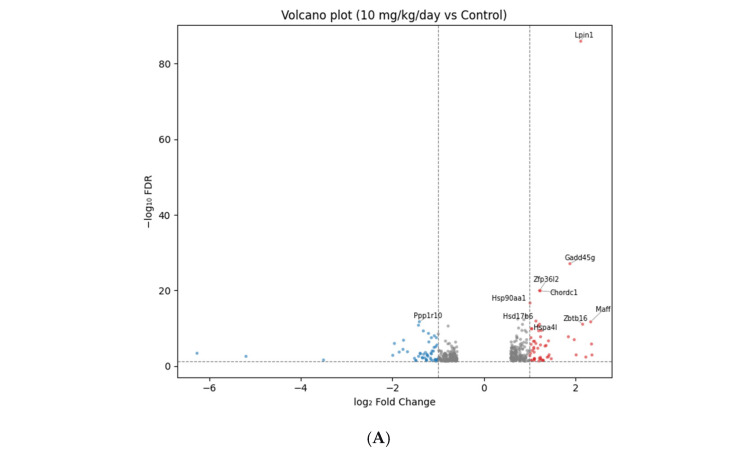

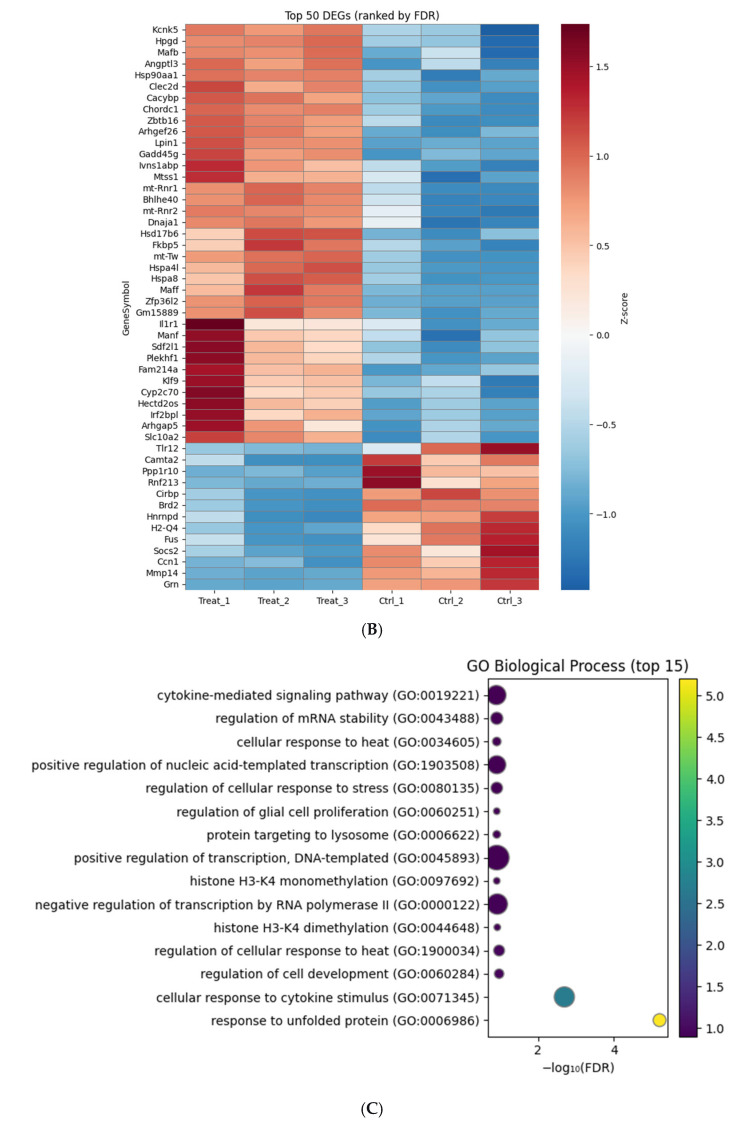

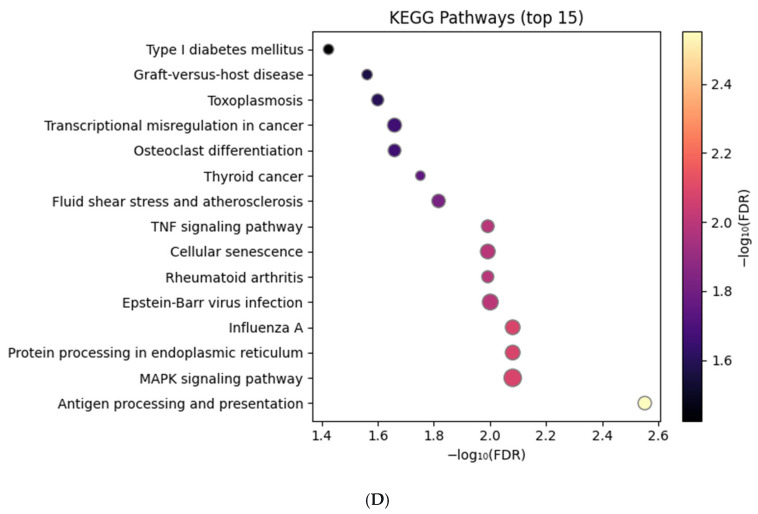
Transcriptomic landscape of murine liver following 28-day oral exposure to BPAF. (**A**) Volcano plot of differentially expressed genes (DEGs) in the 10 mg kg^−1^ day^−1^ BPAF group compared to the control (*n* = 6 per group). Grey dots represent genes with no significant change, while red and blue dots represent significantly up- and down-regulated genes, respectively (thresholds: |log_2_FC| ≥ 1, FDR < 0.05). (**B**) Heatmap of the top 50 DEGs (ranked by adjusted *p*-value) across all dose groups (0.1, 1, and 10 mg kg^−1^ day^−1^), showing Z-score normalized gene expression levels. (**C**) Gene Ontology (GO) enrichment analysis for biological processes. The top 15 significantly enriched terms are displayed based on their −log_10_(*p*-value). (**D**) KEGG pathway enrichment analysis. The node size corresponds to the number of genes mapped to the pathway, and the color intensity represents the significance level (−log_10_(FDR)).

To investigate the systemic hepatic response to BPAF, we performed RNA-seq on mouse liver tissue. Principal component analysis (PCA) showed a clear dose-dependent separation of transcriptomic profiles (Figure 1A). In the high-dose group (10 mg/kg/day), 1247 differentially expressed genes (DEGs) were identified (598 up, 649 down). KEGG pathway enrichment analysis revealed that “PPAR signaling pathway,” “Fatty acid degradation,” and “Glutathione metabolism” were the most significantly perturbed pathways (Figure 1B). Notably, a suite of mitochondrial genes involved in fatty acid β-oxidation (Ppara, Cpt1a, Acadm) and TCA cycle function (Sdha, Sdhb) were significantly downregulated (Figure 1C), suggesting a transcriptional reprogramming away from oxidative phosphorylation.

### 3.2. Metabolomics Identifies Succinate Accumulation and TCA Cycle Arrest

**Figure 2:**
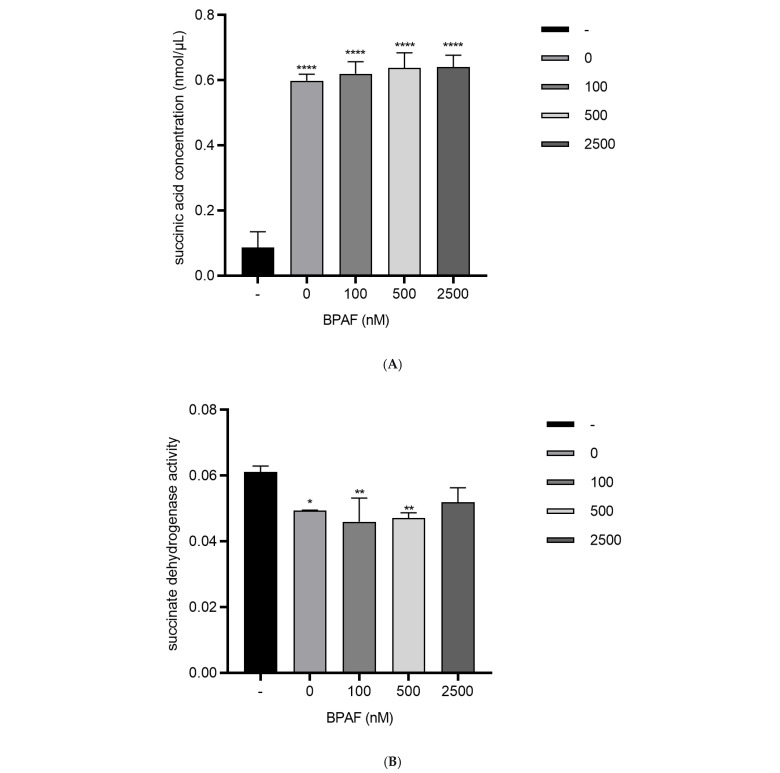

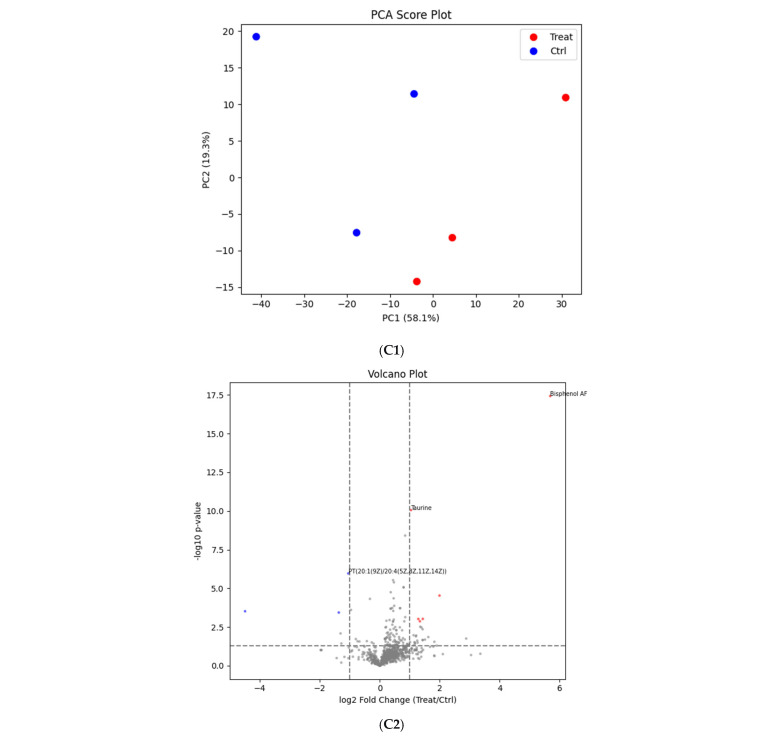

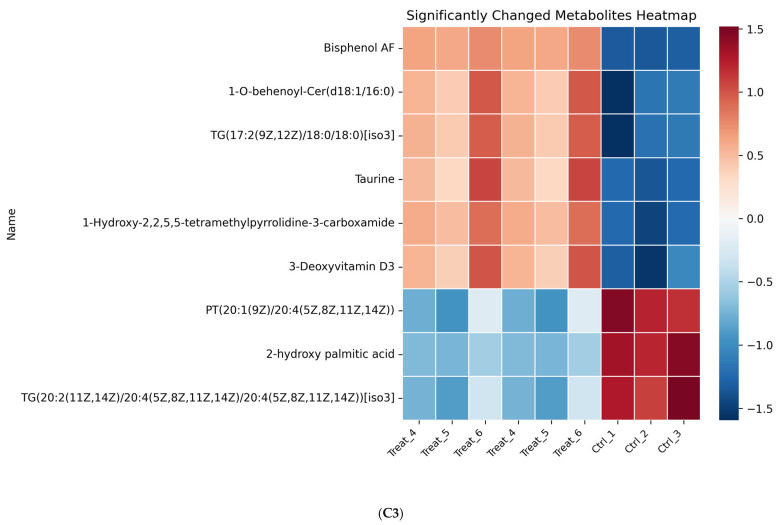
Metabolomic profiling of BPAF-exposed co-cultured hepatocytes and macrophages. (**A**) Dose-dependent increase in extracellular succinate concentration in the supernatant of RAW264.7 macrophages after 48 h of BPAF treatment. Data are mean ± SD (*n* = 3 biological replicates per group). **** *p* < 0.0001 vs. control. (**B**) Succinate dehydrogenase (SDH) activity in RAW264.7 cells following 48 h BPAF exposure. A significant reduction was observed at the highest concentration. Data are mean ± SD (*n* = 3). * *p* < 0.05, ** *p* < 0.01 vs. control. (**C**) Multivariate analysis and differential metabolite screening from non-targeted metabolomics of the co-culture system after 48 h exposure to 2500 nM BPAF. (**C1**) Principal component analysis (PCA) score plot showing a clear separation between treatment (red) and control (blue) groups, indicating a global metabolic shift. (**C2**) Volcano plot displaying fold changes and statistical significance for all detected metabolites. Grey dots represent metabolites with no significant change (|log_2_ fold change| ≤ 1 or FDR ≥ 0.05), while red and blue dots indicate significantly up- and down-regulated metabolites, respectively (|log_2_ fold change| > 1 and FDR < 0.05). (**C3**) Hierarchical clustering heatmap of significantly altered metabolites (rows) across individual samples (columns). Metabolite intensities are Z-score normalized, revealing distinct expression patterns between treatment and control groups.

To determine the functional metabolic consequences of BPAF exposure, we performed non-targeted metabolomics on the co-culture system. Multivariate analysis (PLS-DA) revealed a distinct separation between control and BPAF-treated groups (Figure S1). Pathway impact analysis confirmed that the “TCA cycle” and “Glyoxylate and dicarboxylate metabolism” were among the top impacted pathways (Figure 2C and Figure S3).

A key finding was the significant, dose-dependent accumulation of succinate in the supernatant of RAW264.7 macrophages (Figure 2A). Succinate levels increased nearly 7-fold at the highest concentration (2500 nM, *p* < 0.001). Consistent with this observation, enzymatic assays demonstrated a marked inhibition of succinate dehydrogenase (SDH) activity in BPAF-exposed cells, with a 52.3% reduction at 2500 nM compared to controls (Figure 2B). This metabolic blockade was further supported by the depletion of downstream TCA cycle intermediates, fumarate and malate (Figure 2(C3)).

### 3.3. Integrated Multi-Omics Network Links SDH Inhibition to Lipid Dysregulation

**Figure 3:**
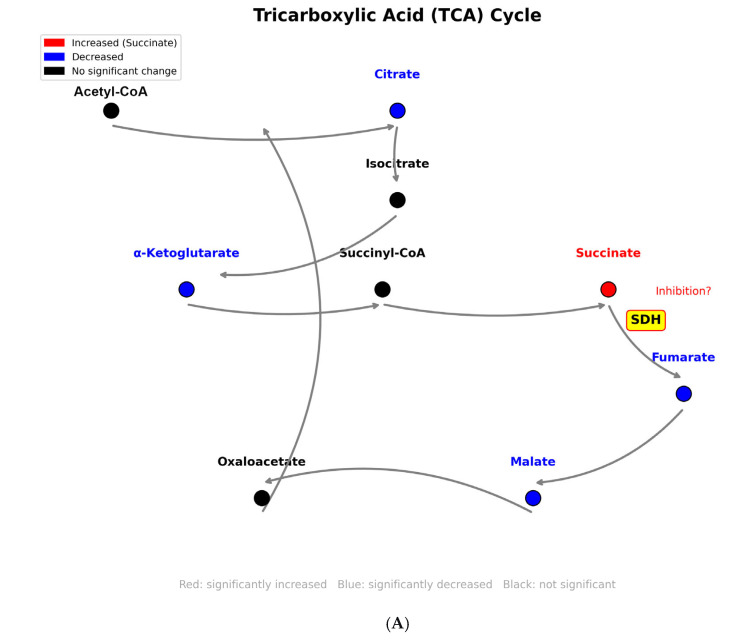

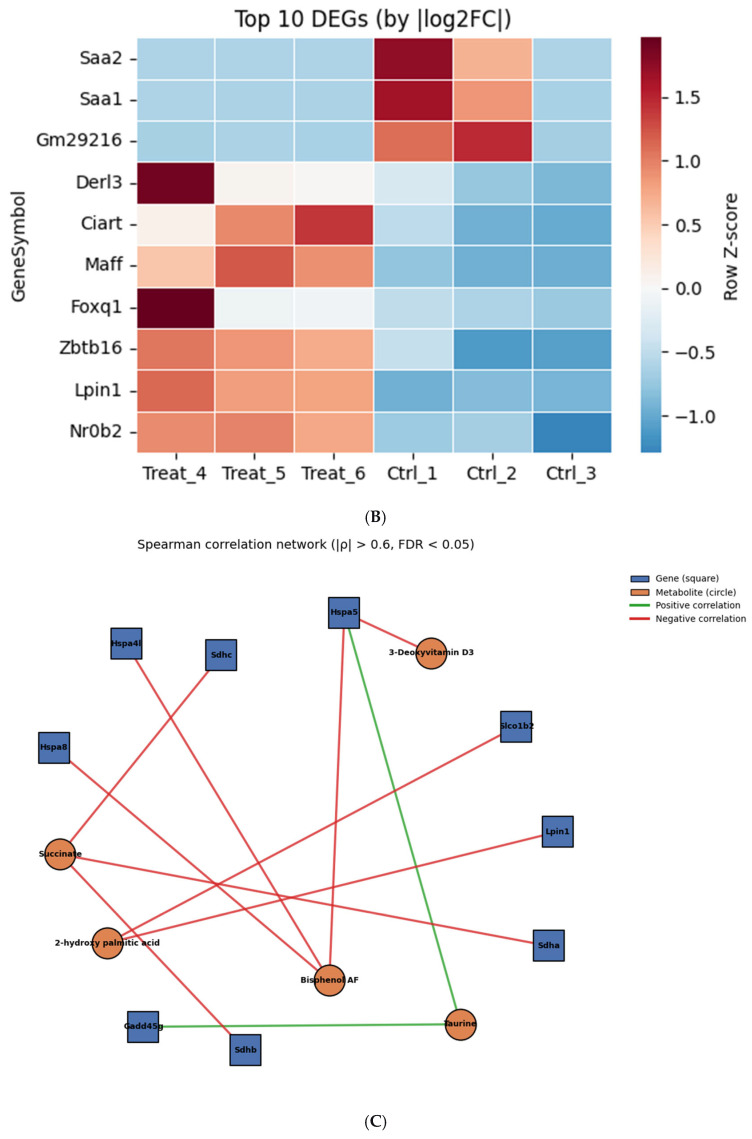
Integrated multi-omics analysis reveals BPAF-induced TCA cycle disruption and mitochondrial dysfunction. (**A**) Schematic of the tricarboxylic acid (TCA) cycle summarizing metabolite changes identified by non-targeted metabolomics in the co-culture system. Solid black arrows indicate normal metabolic flux; the dashed red arrow marks the conversion of succinate to fumarate catalyzed by succinate dehydrogenase (SDH), which was identified as a key metabolic bottleneck. Metabolites significantly increased are labeled in red, and those significantly decreased in blue (VIP > 1, *p* < 0.05). The question mark and the dashed red box indicate that direct SDH inhibition by BPAF remains to be experimentally validated. (**B**) Heatmap of the top 10 differentially expressed genes ranked by absolute log_2_ fold change in livers of mice exposed to 10 mg/kg/day BPAF versus controls (*n* = 6 per group). Each row was Z-score normalized. Red and blue indicate up- and down-regulation, respectively. (**C**) Spearman correlation network constructed from 106 differentially expressed genes (squares) and 9 differentially abundant metabolites (circles) across individual liver samples. Node colors denote the direction of regulation (red: up-regulated; blue: down-regulated). Edges represent significant correlations (green: positive, ρ > 0.6; red: negative, ρ < −0.6) after Benjamini–Hochberg correction (FDR < 0.05). Only 8 significant gene–metabolite pairs met these stringent criteria. A complete list of all gene–metabolite correlations is provided in Table S5.

Correlation network analysis was performed to link transcriptomic and metabolomic alterations. The analysis revealed a strong negative correlation between the accumulation of succinate and the mRNA expression levels of SDH complex subunits (Sdha, Sdhb, Sdhc) (Figure 3A). Furthermore, the downregulation of Ppara and downstream β-oxidation genes correlated with elevated levels of long-chain acylcarnitines, indicating impaired mitochondrial fatty acid import and oxidation (Figure 3B). This integrative network firmly establishes BPAF-induced mitochondrial dysfunction as a central node connecting transcriptional suppression, TCA cycle blockade, and lipid accumulation. A total of 87 differential metabolites were identified (VIP > 1, *p* < 0.05), with the complete list provided in Table S3. Spearman’s rank correlation was used to construct a metabolite–transcript network using the Hmisc version 4.2.0 R package. Only associations with absolute correlation coefficient |ρ| > 0.6 and false discovery rate (FDR) < 0.05 were considered significant. The resulting network was visualised in Cytoscape (v3.9.1, Cytoscape Consortium, San Diego, CA, USA). To link transcriptomic and metabolomic alterations, we performed Spearman correlation network analysis. Note that transcriptomics data were obtained from mouse liver tissues (in vivo), while metabolomics data were obtained from the co-culture system (in vitro) under comparable BPAF exposure conditions (0, 100, 500, 2500 nM). Despite the different biological matrices, we identified consistent pathway-level associations (TCA cycle, fatty acid metabolism). A total of 87 differential metabolites (VIP > 1, *p* < 0.05) and 106 DEGs (|log_2_FC| > 1, FDR < 0.05) were used as input. Spearman’s rank correlation (Hmisc R package (R Foundation for Statistical Computing, Vienna, Austria)) was applied, retaining pairs with |ρ| > 0.6 and FDR < 0.05. The resulting network (Figure 3C) revealed a strong negative correlation between succinate accumulation and SDH complex subunits (Sdha, Sdhb, Sdhc). A complete list of all gene–metabolite correlations is provided in Table S5.

### 3.4. BPAF Induces Oxidative Stress, Inflammation, and Lipid Droplet Formation

**Figure 4:**
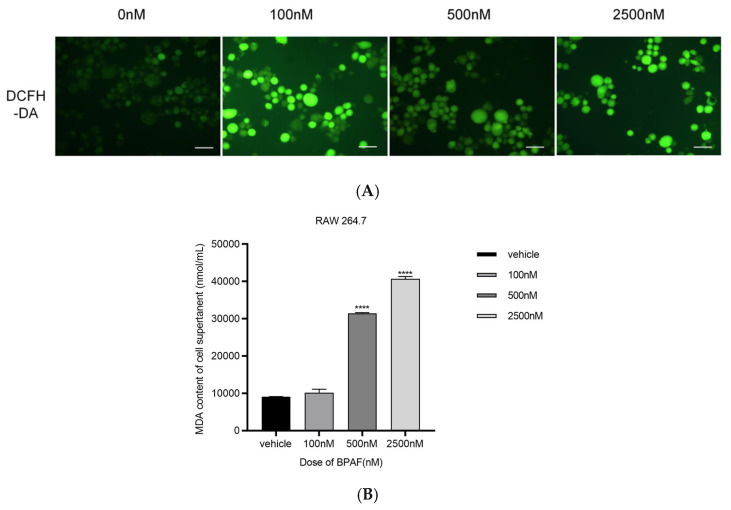

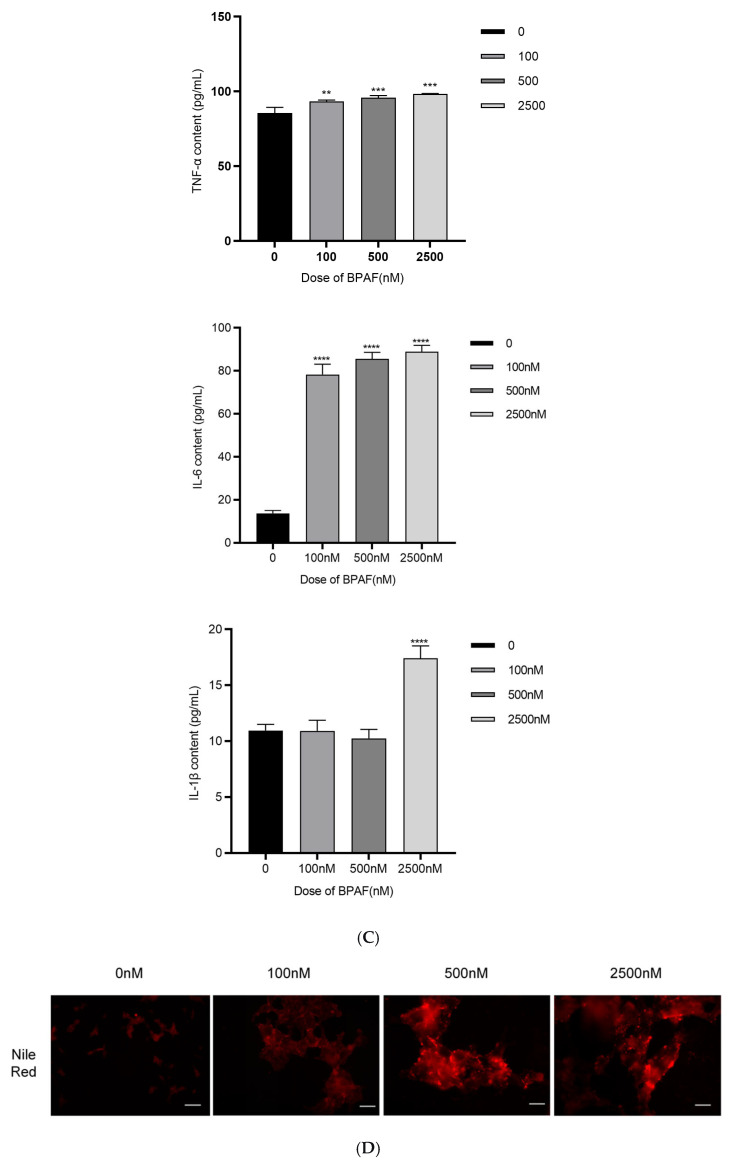

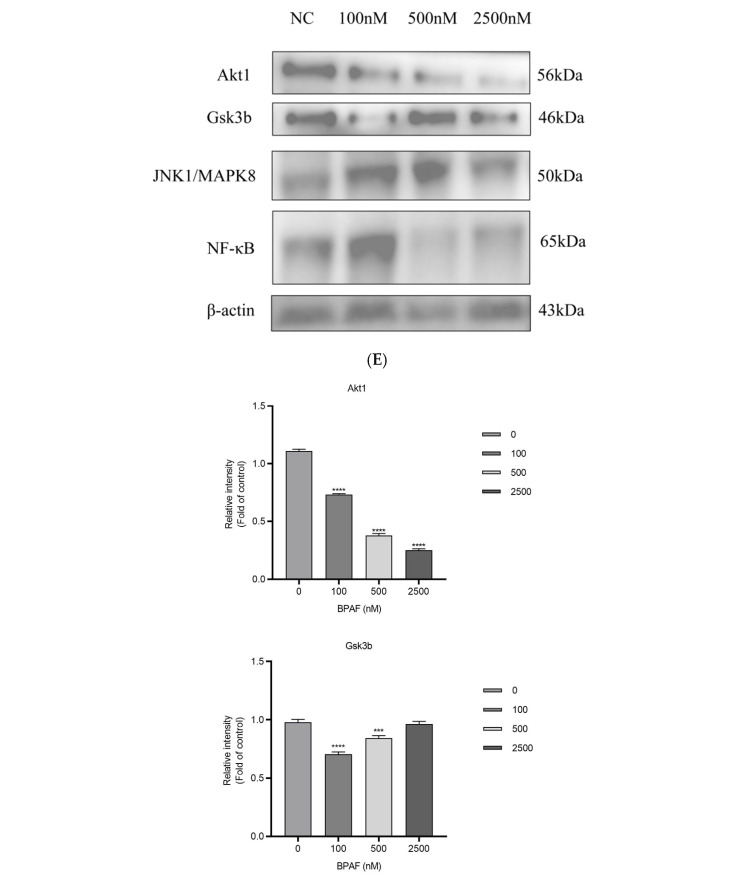

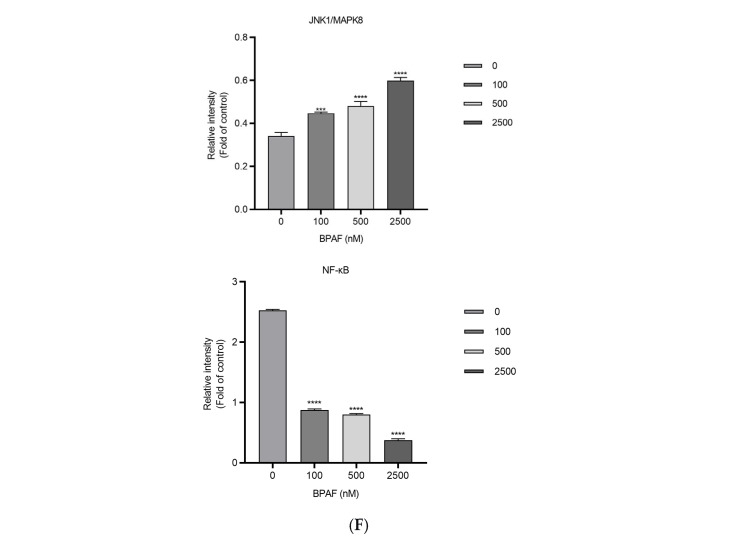
BPAF induces cytotoxicity, inflammation, oxidative stress, and lipid accumulation in vitro. (**A**) Intracellular ROS levels in RAW264.7 cells detected by DCFH-DA fluorescence probe after 24 h of BPAF treatment. Representative fluorescence images are shown. Scale bar = 100 μm; 20× objective. (**B**) Malondialdehyde (MDA) content in the culture supernatant of RAW264.7 cells, indicating the extent of lipid peroxidation. (**C**) Levels of pro-inflammatory cytokines TNF-α, IL-6, and IL-1β in the culture supernatant of RAW264.7 cells after 24 h of BPAF exposure, as determined by ELISA. (**D**) Neutral lipid accumulation in AML12 hepatocytes detected by Nile Red staining after 48 h of BPAF exposure; representative fluorescence images are shown (scale bar = 50 μm; 40× objective). (**E**) Representative Western blot images of Akt1 (56 kDa), GSK3β (46 kDa), JNK1/MAPK8 (50 kDa), NF-κB p65 (65 kDa), and β-actin (43 kDa) in AML12 hepatocytes after 48 h exposure to BPAF (0–2500 nM). (**F**) Densitometric quantification of protein bands normalized to β-actin. Data are mean ± SD from three independent experiments. ** *p* < 0.01, *** *p* < 0.001, **** *p* < 0.0001 vs. control.

Phenotypic validation confirmed the cellular consequences of the observed metabolic disruption. BPAF exposure led to a significant increase in intracellular ROS levels (Figure 4A) and malondialdehyde (MDA) content, a marker of lipid peroxidation (Figure 4B). Pro-inflammatory cytokines, particularly IL-6, were robustly secreted by macrophages in a dose-dependent manner (Figure 4C).

Crucially, Nile Red staining of AML12 hepatocytes revealed a substantial accumulation of neutral lipid droplets following BPAF treatment (Figure 4D). Benchmark dose analysis of the Nile Red fluorescence intensity yielded a BMDL_10_ of 3.2 nM (BMD = 4.8 nM; Table S4). Notably, this value is approximately three orders of magnitude lower than the cytotoxicity BMDL_10_ for AML12 cells (1810 nM; Table S4), confirming that lipid accumulation occurs at sub-cytotoxic concentrations. As shown in Figure 4E,F, BPAF exposure led to decreased protein expression of Akt1 and GSK3β, as well as increased expression of JNK1/MAPK8 and NF-κB p65, consistent with the transcriptomic findings. Benchmark dose (BMD) analysis was performed for all concentration–response data; complete BMD results are provided in Table S4.

## 4. Discussion

In this study, we employed an integrated multi-omics approach to delineate the mechanism of BPAF-induced hepatic lipotoxicity. Succinate accumulation is not merely a biomarker of TCA cycle dysfunction; it acts as a signaling molecule (“pseudohypoxia”) that stabilizes HIF-1α, promoting inflammation and fibrosis [9,10]. Our results demonstrate that BPAF disrupts mitochondrial homeostasis by inhibiting succinate dehydrogenase (SDH), a key enzyme complex linking the TCA cycle to the electron transport chain [11]. This inhibition leads to a metabolic bottleneck characterized by succinate accumulation, oxidative stress, and a transcriptional shift that favors lipid accumulation over fatty acid oxidation.

The convergence of transcriptomic and metabolomic data on the TCA cycle provides robust evidence for SDH as a specific molecular target of BPAF. While prior studies have noted BPAF-induced steatosis and PPAR disruption, our study is the first to mechanistically link these phenotypes to SDH inhibition. Succinate accumulation is not merely a biomarker of TCA cycle dysfunction; it acts as a signaling molecule (“pseudohypoxia”) that stabilizes HIF-1α, promoting inflammation and fibrosis [12]. A parallel study from our group further identified VDAC1 as an upstream mediator of BPAF-induced succinate accumulation and p38 MAPK/NF-κB-driven inflammation, providing additional mechanistic depth to the SDH dysfunction observed herein [13]. The observed increase in IL-6 secretion by macrophages in our co-culture system aligns with this succinate-driven inflammatory response.

While the present manuscript was under evaluation, Wang et al. (2025) [14] independently reported that BPAF induces hepatic steatosis through succinate–SUCNR1-mediated macrophage–hepatocyte interactions. Their adverse outcome pathway framework highlighted succinate as a key molecular initiating event, which is in excellent agreement with our core findings. However, our study substantially extends this mechanistic model in four ways: (i) we provide direct, dose-dependent evidence of a 52% reduction in SDH enzymatic activity and a concomitant 7-fold succinate accumulation in a co-culture system, demonstrating that the metabolic block is quantitative and cell-type specific; (ii) we integrate matched transcriptomics and metabolomics from consistent exposure conditions to construct a correlation network, revealing a strong negative association between succinate accumulation and Sdha/Sdhb expression; (iii) our benchmark dose analysis establishes a BMDL_10_ for lipid accumulation (3.2 nM) that is orders of magnitude below the cytotoxicity threshold, offering a potency metric suitable for risk assessment; and (iv) we delineate a transcriptional signature involving PPARα and its downstream β-oxidation targets as a likely consequence, not merely a correlate, of SDH dysfunction.

The downregulation of Ppara and its target genes involved in mitochondrial β-oxidation (e.g., Cpt1a, Acadm) explains the observed shift toward lipid storage. When mitochondrial fatty acid oxidation is impaired due to SDH inhibition and reduced electron transport chain flux, fatty acids are redirected toward esterification and storage as lipid droplets [15]. This mechanism provides a coherent explanation for the steatotic phenotype observed in Nile Red staining and is consistent with emerging evidence linking environmental mitochondrial toxicants to the pathogenesis of non-alcoholic fatty liver disease (NAFLD) [16].

The use of a hepatocyte–macrophage co-culture model was instrumental in capturing the crosstalk between parenchymal cells and immune cells [17]. The robust succinate accumulation observed in the macrophage compartment suggests that Kupffer cells may be early sensors of BPAF-induced metabolic stress, releasing pro-inflammatory signals that exacerbate hepatocyte dysfunction [13,18]. This model, while simplified, provides a more physiologically relevant context than monoculture for studying liver toxicants.

### Relevance of Exposure Concentrations to Human Health

The translation of experimental exposure levels to human risk assessment is critical. The mouse doses (0.1–10 mg/kg/day) correspond to human equivalent doses (HED) of approximately 0.008–0.81 mg/kg/day based on body surface area scaling. The lowest dose (HED ≈ 0.008 mg/kg/day) is within one order of magnitude of estimated daily intake from human biomonitoring [19] (mean plasma BPAF 0.073 ng/mL, ~0.22 nM; estimated daily intake 0.0048–0.75 μg/kg bw/day).

In vivo doses. The mouse doses employed (0.1–10 mg/kg/day for 28 days) span from environmentally relevant to overtly toxic exposure levels. Based on body surface area-based allometric scaling [20], these doses correspond to human equivalent doses (HED) of approximately 0.008–0.81 mg/kg/day. For the lowest dose (0.1 mg/kg/day, HED ≈ 0.008 mg/kg/day), this is within one order of magnitude of the estimated daily intake (EDI) derived from human biomonitoring data. Jin et al. (2018) reported a mean plasma BPAF concentration of 0.073 ng/mL in Chinese adults, with estimated EDI values for total bisphenols in the range of 0.0048–0.75 μg/kg bw/day [19]. Although our administered doses are partly above typical human dietary exposures, they are relevant for occupational or high-exposure scenarios and are consistent with dose ranges used in recent in vivo studies of BPAF-induced metabolic disruption [6,14]. Pharmacokinetic studies indicate that orally administered BPAF is rapidly absorbed and extensively conjugated, with a relatively low oral bioavailability of approximately 3–6% in mice [21], implying that the parent BPAF reaching target tissues in our animals is considerably lower than the administered dose.

In vitro concentrations. For in vitro concentrations (100–2500 nM; 0.034–0.84 μg/mL), the lower end is two to three orders of magnitude above typical general population serum levels (~0.22 nM) [19]. However, this range provides a safety margin accounting for bioaccumulation in lipid-rich tissues, interspecies differences, and local tissue concentrations [22]. Notably, the BMDL_10_ for lipid accumulation (3.2 nM) is substantially lower than the cytotoxicity threshold (>1800 nM) [23], demonstrating that BPAF perturbs lipid homeostasis at sub-cytotoxic concentrations that are closer to human internal exposures [2]. These findings suggest that even low-level BPAF exposure may contribute to metabolic dysfunction, consistent with the concept of non-monotonic dose responses for endocrine disruptors [23].

Co-culture model considerations. The Transwell co-culture system employed in this study, in which RAW264.7 macrophages and AML12 hepatocytes are physically separated by a semi-permeable membrane (3.0 µm pore size), permits paracrine signaling via soluble factors and extracellular vesicles while preventing direct cell-to-cell contact. This configuration partially recapitulates the in vivo hepatic sinusoid, where Kupffer cells (liver-resident macrophages) and hepatocytes engage in bidirectional communication essential for metabolic homeostasis and inflammatory responses [24]. In the present study, the robust succinate accumulation observed in the macrophage compartment following BPAF exposure suggests that macrophages may function as early sensors of xenobiotic-induced mitochondrial stress, releasing soluble mediators that secondarily impair hepatocyte lipid handling—a mechanism that is supported by the recent identification of the succinate–SUCNR1 signaling axis in BPAF-induced steatosis [14]. Nevertheless, this simplified model does not fully replicate the complexity of the native liver microenvironment. Key limitations include the absence of three-dimensional architecture, physiological oxygen zonation, hemodynamic shear forces, and contributions from other liver-resident cell types such as hepatic stellate cells (fibrogenesis) and liver sinusoidal endothelial cells (barrier function). Furthermore, the enterohepatic axis—including the potential role of gut-derived microbial metabolites in modulating hepatic BPAF toxicity—is not captured in this in vitro system. Future studies employing liver organoids, microphysiological liver-on-a-chip platforms, or in vivo co-exposure models are warranted to validate the translational relevance of the macrophage–hepatocyte crosstalk observed herein.

Limitations: Histological examination of liver tissues (e.g., H&E or Oil Red O staining) was not performed, as samples were prioritized for transcriptomics. Future studies should include histopathology to visually confirm steatosis. Additionally, while we used well-characterized murine cell lines (AML12, RAW264.7) for high-throughput screening, validation in human primary hepatocytes or liver organoids is warranted to confirm translational relevance.

## 5. Conclusions

In summary, this study suggests that BPAF exposure is associated with succinate dehydrogenase dysfunction and TCA cycle disruption in hepatic and macrophage models. By integrating transcriptomics and metabolomics, we provide evidence consistent with the hypothesis that BPAF exposure may contribute to mitochondrial dysfunction, oxidative stress, and a metabolic shift toward hepatic lipid accumulation. These findings advance our understanding of how environmental bisphenols may be linked to metabolic liver disease and underscore the value of multi-omics approaches in uncovering unanticipated toxicological mechanisms.

## Data Availability

The RNA-seq data generated in this study have been deposited in the National Genomics Data Center (NGDC) repository under accession number OMIX010050. Additional metabolomics data and analysis scripts are available upon reasonable request from the corresponding author.

## Supplementary Materials

The following supporting information can be downloaded at: https://www.mdpi.com/article/10.3390/metabo16070440/s1, Supplementary Methods S1: Detailed Cell Culture Conditions; Supplementary Methods S2: Transwell Co-culture System Setup; Supplementary Methods S3: Metabolomics Quality Control Procedures; Supplementary Methods S4: Benchmark Dose Modeling Parameters; Supplementary Methods S5: Multi Omics Integration: Correlation Network Analysis; Supplementary Methods S6: Study Design Flowchart; Figure S1: PLS-DA score plot of transcriptomic profiles; Figure S2: Schematic diagram of the experimental design; Figure S3: Pathway enrichment analysis of differential metabolites categorized by chemical class in BPAF-exposed AML12 hepatocytes and RAW264.7 macrophages; Table S1: Complete list of qRT-PCR primers; Table S2: Primary and secondary antibodies used for Western blot analysis; Table S3: Complete list of differential metabolites; Table S4: Complete benchmark dose (BMD) results for all endpoints; Table S5: Significant gene–metabolite correlations from multi-omics integration.
