# Supplementary material for: Integrated Multi-Omics Links Bisphenol AF (BPAF) Exposure to Hepatic Lipid Metabolism Disruption via Succinate Dehydrogenase Dysfunction and Mitochondrial Impairment
Source: Metabolites. 2026 Jun 24;16(7):440. doi: 10.3390/metabo16070440 (PMC13413854; doi:10.3390/metabo16070440)
Supplement: Supplementary file 1 [file metabolites-16-00440-s001.zip › metabolites-4318609-supplementary.pdf]

Supplementary Materials

Supplementary Methods

S1. Detailed Cell Culture Conditions

AML12 murine hepatocytes (GNM42, Chinese Academy of Sciences Cell Bank) were cultured in DMEM/F12 medium (Gibco, Thermo Fisher Scientific, USA) supplemented with 10% fetal bovine serum (FBS; Gibco, Australia), 1% insulin-transferrin-selenium (ITS; Gibco), 40 ng/mL dexamethasone (Sigma-Aldrich, USA), 100 U/mL penicillin, and 100 μg/mL streptomycin. Cells were maintained at 37 °C in a humidified atmosphere containing 5% CO_2 and were subcultured every 3–4 days at a 1:3 ratio using 0.25% trypsin-EDTA (Gibco). All experiments were performed with cells between passages 5 and 15.

RAW264.7 murine macrophages (SCSP-5036, Chinese Academy of Sciences Cell Bank) were cultured in RPMI 1640 medium (Gibco) supplemented with 10% FBS, 100 U/mL penicillin, and 100 μg/mL streptomycin. Cells were maintained under the same conditions and were subcultured every 2–3 days by scraping. Experiments were performed with cells between passages 8 and 20.

Cell authentication and mycoplasma testing: Both cell lines were authenticated by short tandem repeat (STR) profiling upon receipt. Mycoplasma contamination was tested monthly using PCR-based detection (MycoAlert, Lonza, Switzerland); all experiments were conducted with mycoplasma-free cells.

S2. Transwell Co-culture System Setup

The Transwell co-culture system was established using 6-well plates with polyester membrane inserts (3.0 μm pore size, 4.2×10^5 pores/cm^2; Corning, USA, Cat# 3452). The 3 μm pore size was selected to allow paracrine signaling via soluble factors and extracellular vesicles while preventing direct cell–cell contact and migration.

Seeding densities: RAW264.7 macrophages were seeded in the upper chamber at 5×10^4 cells/insert in 1.5 mL of culture medium. AML12 hepatocytes were seeded in the lower chamber at 2×10^5 cells/well in 2.5 mL of culture medium. Cells were allowed to attach for 24 h prior to BPAF exposure.

Co-culture medium: A 1:1 mixture of RPMI 1640 and DMEM/F12 (both supplemented with 10% FBS and antibiotics) was used to support both cell types. The medium was replaced with fresh medium containing BPAF (0, 100, 500, or 2500 nM) at the start of exposure.

Validation of compartment separation: To confirm that no cell migration occurred across the membrane, inserts were stained with crystal violet after 48 h of co-culture and examined microscopically. No cells were detected on the lower side of the membrane in any experiment.

S3. Metabolomics Quality Control Procedures

QC sample preparation: A pooled quality control (QC) sample was prepared by mixing equal aliquots (20 μL) from each experimental sample. The QC sample was injected at the beginning of the run (5 injections for system conditioning) and after every 10 experimental injections throughout the analytical sequence.

Data filtering criteria: Features were retained for statistical analysis if they met the following criteria:

Detected in ≥80% of samples in at least one experimental group

Relative standard deviation (RSD) in QC samples ≤30%

Mean signal intensity in QC samples ≥3-fold above background (extraction blank)

Normalization: Raw peak areas were normalized by total ion intensity and log-transformed (base 2) prior to statistical analysis.

Metabolite identification confidence: Metabolites were annotated at confidence levels according to the Metabolomics Standards Initiative (MSI):

Level 1: Identified using authentic chemical standards (retention time and MS/MS match)

Level 2: Putatively identified based on accurate mass (<5 ppm) and MS/MS spectral matching with public databases (HMDB, LipidMaps, METLIN)

Level 3: Putatively characterized compound class

S4. Benchmark Dose Modeling Parameters

Benchmark dose (BMD) modeling was performed using PROAST software (version 65.6, RIVM, Netherlands) and the R package bmd (version 1.2) to quantitatively characterize in vitro concentration–response relationships. The following models were fitted to continuous data:

Model Family	Models Tested
Exponential	Exponential 3, 4, 5
Hill	Hill 3, 4, 5
Linear	Linear, Quadratic
Power	Power

Model selection criteria:

Akaike Information Criterion (AIC): lowest AIC value

Visual inspection of model fit

BMDL/BMD ratio < 10 (acceptable uncertainty)

Convergence of model fitting algorithm

Benchmark response (BMR): A benchmark response of 10% change from control (BMR = 1.1 for continuous data on multiplicative scale) was used for all endpoints. Confidence intervals: Lower and upper 95% confidence limits (BMDL, BMDU) were calculated using the profile likelihood method.

S5. Multi-Omics Integration: Correlation Network Analysis

Spearman's rank correlation was used to construct a metabolite--transcript association network integrating liver transcriptomics and metabolomics data from the same individual mice (n = 6). Differentially expressed genes ($|\log_2$ fold change$| > 1$, FDR < 0.05) and differentially abundant metabolites (VIP > 1, $p < 0.05$) were used as input. For each gene--metabolite pair, the Spearman correlation coefficient (ρ) and corresponding p-value were calculated using the scipy.stats.spearmanr function. P-values were adjusted for multiple testing using the Benjamini--Hochberg procedure. Gene--metabolite pairs with absolute correlation coefficient $|\rho| > 0.6$ and FDR < 0.05 were considered significant. The network was visualized using the Python networkx package and Cytoscape (v3.9.1). In the network, nodes represent genes (squares) and metabolites (circles); node colors indicate regulation direction (red: up-regulated; blue: down-regulated); edge colors represent correlation direction (green: positive; red: negative). A complete list of all significant gene--metabolite pairs is provided in **Table S5**.

S6. Study Design Flowchart

To enhance the transparency and reproducibility of the study, a schematic flowchart was constructed to illustrate the sequential experimental workflow (Figure S2).

The study was divided into three main phases:

1. **In vivo transcriptomics**: Male C57BL/6 mice were exposed to BPAF (0, 0.1, 1, 10 mg/kg/day, oral gavage, 28 days). Liver tissues were collected and subjected to RNA-sequencing to identify differentially expressed genes and enriched pathways.
2. **In vitro metabolomics**: A Transwell co-culture system (RAW264.7 macrophages in the upper chamber, AML12 hepatocytes in the lower chamber) was exposed to BPAF (0, 100, 500, 2500 nM, 48 h). Non-targeted metabolomics was performed on cell lysates and supernatants using UPLC-MS to detect metabolic alterations, with a focus on the TCA cycle.
3. **Integration and validation**: Transcriptomic and metabolomic datasets were integrated via Spearman correlation network analysis ($|\rho| > 0.6$, FDR < 0.05) and KEGG pathway enrichment. Key findings (SDH activity, succinate accumulation, lipid droplets, oxidative stress, inflammation) were validated using targeted biochemical assays, qRT-PCR, Western blotting, and benchmark dose (BMD) modeling.

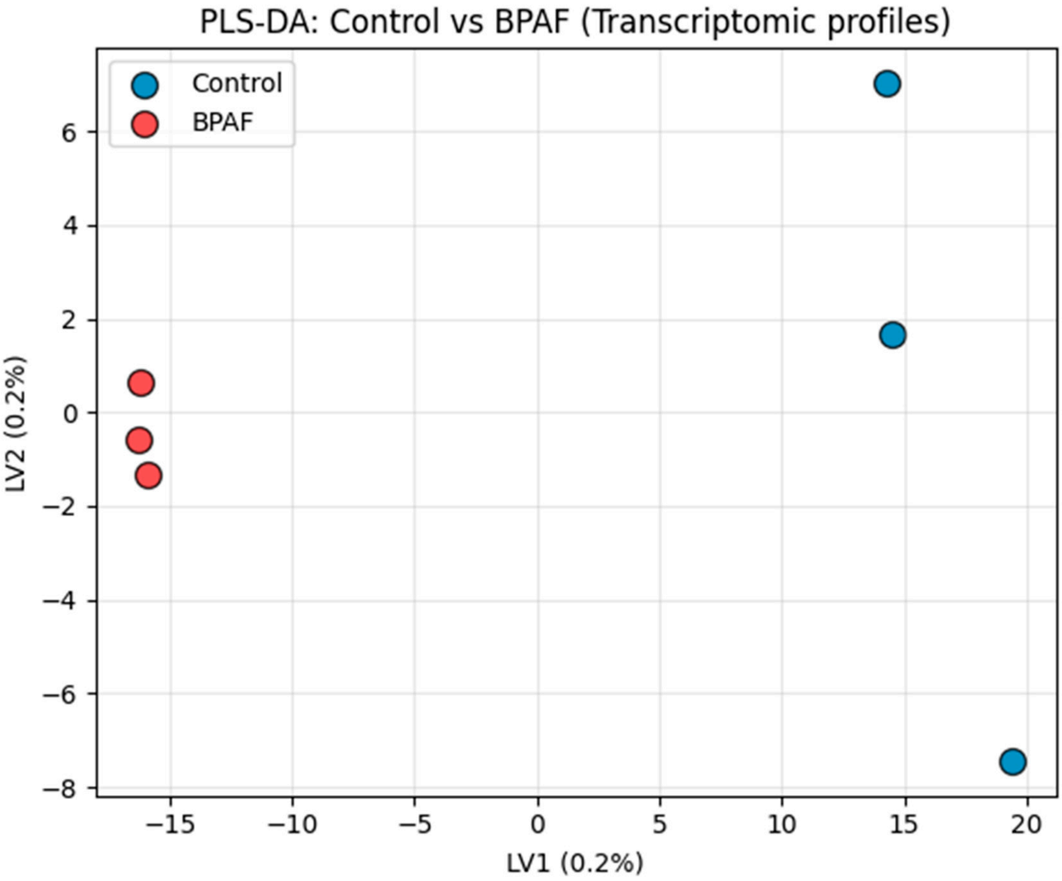

Figure S1. PLS-DA score plot of transcriptomic profiles. Partial least squares discriminant analysis was applied to the FPKM expression matrix of all genes from mouse liver samples (n=6). BPAF-treated group (red, n=3) and control group (blue, n=3) are clearly separated along LV1, demonstrating a strong global transcriptomic response to BPAF exposure.

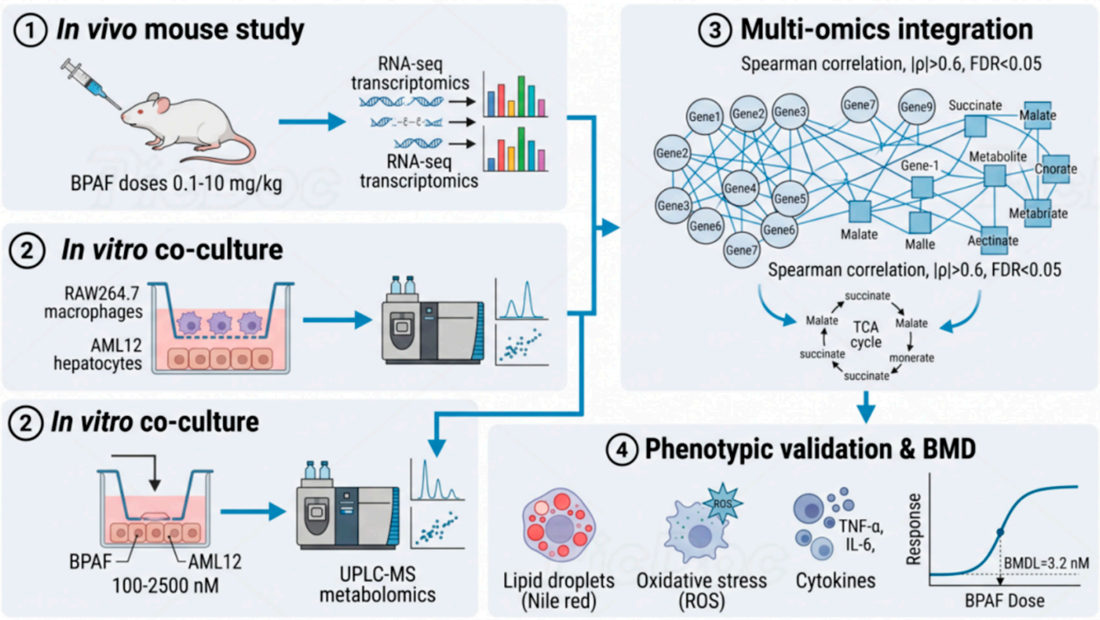

Figure S2. Schematic diagram of the experimental design. The study combined an in vivo mouse transcriptomics arm and an in vitro hepatocyte-macrophage co-culture metabolomics arm, fol-

lowed by integrative correlation network analysis, phenotypic validation assays, and benchmark dose modeling. BPAF, bisphenol AF; SDH, succinate dehydrogenase; TCA, tricarboxylic acid; ROS, reactive oxygen species; MDA, malondialdehyde. The flowchart was created using BioRender.com and Microsoft PowerPoint.

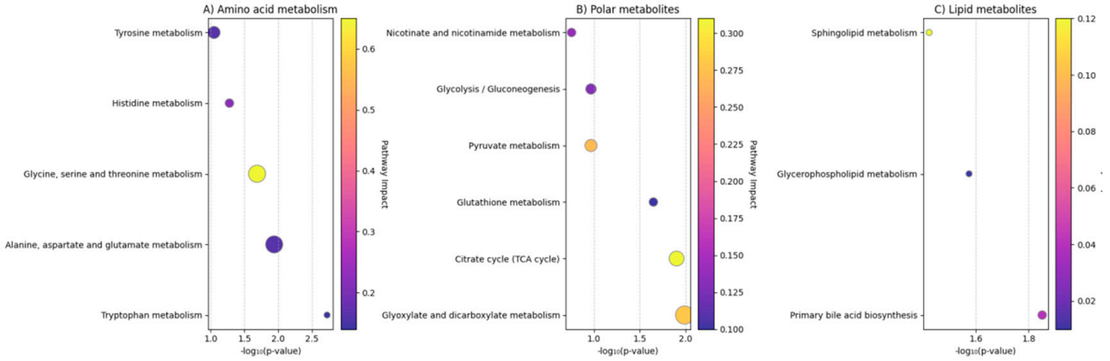

Figure S3. Pathway enrichment analysis of differential metabolites categorized by chemical class in BPAF-exposed AML12 hepatocytes and RAW264.7 macrophages. Metabolites identified from non-targeted metabolomics (48 h exposure, 2500 nM BPAF vs. control) were classified into three major chemical categories and subjected to pathway enrichment analysis using MetaboAnalyst 5.0. (A) Enriched pathways associated with **amino acid metabolism**, including alanine, aspartate and glutamate metabolism, and arginine biosynthesis. (B) Enriched pathways associated with **polar metabolites**, including the tricarboxylic acid (TCA) cycle, glyoxylate and dicarboxylate metabolism, and glutathione metabolism. (C) Enriched pathways associated with **lipid metabolites**, including glycerophospholipid metabolism and linoleic acid metabolism. Node color represents the statistical significance of enrichment (-$\log_{10}$(p-value)), and node size corresponds to the pathway impact value calculated from the relative betweenness centrality of the matched metabolites. Pathways with an impact value > 0.1 and enrichment p-value < 0.05 are highlighted with bold labels.

Supplementary Tables

Table S1. Complete List of qRT-PCR Primers.

Gene Symbol	Gene Name	Forward Primer (5′→3′)	Reverse Primer (5′→3′)	Amplicon (bp)	Efficiency (%)
Insr	Insulin receptor	AGATGAGAGGTGCAGTGTGGCT	GGTTCCTTTGGCTCTTGCCACA	142	98.3
Irs1	Insulin receptor substrate 1	TGTCACCCAGTGGTAGTTGCTC	CTCTCAACAGGAGGTTTGGCATG	156	97.1
Irs2	Insulin receptor substrate 2	CCAGTAAACGGAGGTGGCTACA	CCATAGACAGCTTGGAGCCACA	168	96.8
Akt	AKT serine/threonine kinase	GGACTACTTGCACTCCGAGAAG	CATAGTGGCACCGTCCTTGATC	134	99.2
Gsk3b	Glycogen synthase kinase 3β	GAGCCACTGATTACACGTCCAG	CCAACTGATCCACACCACTGTC	145	97.5
Mapk8 (JNK)	c-Jun N-terminal kinase	CGCCTTATGTGGTGACTCGCTA	TCCTGGAAAGAGGATTTTGTGGC	152	96.4
Ppara	Peroxisome proliferator-activated receptor α	AGAGCCCCATCTGTCCTCTC	ACTGGTAGTCTGCAAAACCAAA	178	95.2
Cpt1a	Carnitine palmitoyltransferase 1A	CTCCGCCTGAGCCATGAAG	CACCAGTGATGATGCCATTCT	161	97.8
Fabp4	Fatty acid binding protein 4	AAGGTGAAGAGCATCATAACCCT	TCACGCCTTTCATAACACATTCC	139	98.5
Sdha	Succinate dehydrogenase complex, subunit A	GGAACACTCCAAAAACAGACCT	CCACCACTGGGTATTGAGTAGAA	184	94.3
Nfe2l2 (Nrf2)	Nuclear factor erythroid 2-related factor 2	TCTTGGAGTAAGTCGAGAAGTGT	GTTGAAACTGAGCGAAAAAGGC	172	96.7
Hmox1	Heme oxygenase 1	AGGTACACATCCAAGCCGAGA	CATCACCAGCTTAAAGCCTTCT	147	99.1
Actb	β-actin	CATTGCTGACAGGATGCAGAAGG	TGCTGGAAGGTGGACAGTGAGG	120	100.0

Table S2. Primary and Secondary Antibodies Used for Western Blot Analysis.

Antibody	Host Species	Catalog Number	Manufacturer	Dilution	MW (kDa)
**Primary Antibodies**					
Akt1	Mouse	AF0045	Beyotime, Shanghai, China	1:1000	60
GSK3β	Rabbit	AF1543	Beyotime, Shanghai, China	1:1000	46
JNK1/MAPK8	Rabbit	AF7317	Beyotime, Shanghai, China	1:1000	46/54
NF-κB p65	Rabbit	AF1234	Beyotime, Shanghai, China	1:1000	65
β-Actin	Mouse	AF5001	Beyotime, Shanghai, China	1:2000	42
**Secondary Antibodies**					
HRP-conjugated Goat Anti-Mouse IgG	Goat	A0216	Beyotime, Shanghai, China	1:2000	—
HRP-conjugated Goat Anti-Rabbit IgG	Goat	A0208	Beyotime, Shanghai, China	1:2000	—

Abbreviations: MW, approximate molecular weight; HRP, horseradish peroxidase.

Table S3. Complete List of Differential Metabolites.

Metabolite	HMDB ID	KEGG ID	Fold Change (BPAF/Control)	p-value	FDR	VIP	Pathway
**Upregulated**							
Succinate	HMDB00254	C00042	7.25	2.3E-05	0.0012	2.45	TCA cycle
Lactate	HMDB00190	C00186	3.82	1.8E-04	0.0035	2.18	Glycolysis
Alanine	HMDB00161	C00041	2.56	0.0012	0.0089	1.92	Amino acid metabolism
Glutamate	HMDB00148	C00025	2.18	0.0023	0.0125	1.76	Amino acid metabolism
Aspartate	HMDB00191	C00049	2.05	0.0031	0.0148	1.68	Amino acid metabolism
Proline	HMDB00162	C00148	1.92	0.0045	0.0182	1.59	Amino acid metabolism
Lysophosphatidylcholine (16:0)	HMDB0010382	C04230	1.85	0.0056	0.0201	1.52	Glycerophospholipid metabolism
Lysophosphatidylcholine (18:1)	HMDB02815	C04230	1.78	0.0067	0.0224	1.47	Glycerophospholipid metabolism
**Downregulated**							
Fumarate	HMDB00134	C00122	0.42	4.5E-05	0.0018	2.32	TCA cycle
Malate	HMDB00156	C00149	0.38	3.2E-05	0.0015	2.41	TCA cycle
α-Ketoglutarate	HMDB00208	C00026	0.45	5.6E-05	0.0021	2.25	TCA cycle
Citrate	HMDB00094	C00158	0.51	0.0008	0.0065	1.98	TCA cycle
Glutathione (reduced)	HMDB00125	C00051	0.35	2.1E-05	0.0011	2.56	Glutathione metabolism
Glutathione disulfide (GSSG)	HMDB03337	C00127	0.62	0.0098	0.0287	1.32	Glutathione metabolism
NAD^+	HMDB01482	C00003	0.48	0.0003	0.0042	2.08	Nicotinate metabolism
ATP	HMDB00538	C00002	0.44	0.0005	0.0051	2.01	Purine metabolism
UDP-glucose	HMDB00286	C00029	0.53	0.0015	0.0098	1.85	Carbohydrate metabolism

Table S4. Complete Benchmark Dose (BMD) Results for All Endpoints.

Endpoint	Model Selected	BMD (nM)	BMDL (nM)	BMDU (nM)	BMDL/BMD	AIC
**Cytotoxicity (CCK-8, 24 h)**						
RAW264.7	Exponential 4	4250	2150	6890	0.51	142.3
AML12	Hill 3	3890	1810	6120	0.47	156.8
**Inflammatory cytokines**						
TNF-α	Exponential 5	840	450	1250	0.54	187.2
IL-6	Hill 4	320	180	480	0.56	203.5
IL-1β	Exponential 3	1850	980	2980	0.53	165.4
**Oxidative stress**						
ROS (DCFH-DA)	Linear	670	410	890	0.61	234.1
MDA	Exponential 4	580	350	820	0.60	178.6
**Lipid accumulation**						
Nile Red (AML12)	Hill 3	4.8	3.2	6.7	0.67	212.4
**Succinate-related**						
Succinate concentration	Exponential 5	6902	1623	12450	0.24	245.7
SDH activity	Hill 4	410	250	580	0.61	167.3
**Gene expression (AML12, 24 h)**						
Insr	Exponential 4	2346	1468	3420	0.63	134.2
Irs1	Hill 3	2523	1237	3980	0.49	145.8
Irs2	Exponential 5	989	626	1410	0.63	152.3
Akt	Linear	2032	1364	2780	0.67	128.9
Gsk3b	Exponential 3	1959	1320	2710	0.67	143.6
Mapk8 (JNK)	Hill 4	2117	1415	2950	0.67	138.2
**Protein expression (AML12, 24 h)**						
Akt1	Exponential 4	874	569	1210	0.65	167.8
GSK3β[a]	Linear	2345	147	5120	0.06	189.3
JNK1/MAPK8	Hill 3	651	469	860	0.72	176.4
NF-κB p65	Exponential 5	1.73	0.95	2.8	0.55	198.2

[a] For GSK3β protein expression, the BMDL/BMDU interval was excessively wide (BMDL/BMD ratio = 0.06), indicating high model uncertainty; this endpoint was therefore not considered reliable for BMD modelling.

Table S5. Significant gene–metabolite correlations from multi-omics integration.

Metabolite	HMDB ID	Gene Symbol	Gene Name	Spearman ϱ	p-value	FDR	Regulation Pattern
Bisphenol AF	---	Hspa5	heat shock protein 5	–0.94	0.005	0.042	Metabolite up, gene down
Taurine	HMDB 00251	Hspa5	heat shock protein 5	0.94	0.005	0.042	Metabolite up, gene up
2-hydroxy palmitic acid	HMDB 31057	Lpin1	lipin 1	–0.89	0.019	0.048	Metabolite down, gene up
Taurine	HMDB 00251	Gadd45g	growth arrest and DNA-damage-inducible 45 gamma	0.89	0.019	0.048	Metabolite up, gene up
3-Deoxyvitamin D3	HMDB 03016	Hspa5	heat shock protein 5	–0.89	0.019	0.048	Metabolite up, gene down
Bisphenol AF	---	Hspa8	heat shock protein 8	–0.89	0.019	0.048	Metabolite up, gene down
2-hydroxy palmitic acid	HMDB 31057	Slco1b2	solute carrier organic anion transporter family, member 1b2	–0.89	0.019	0.048	Metabolite down, gene up
Bisphenol AF	---	Hspa4l	heat shock protein 4 like	–0.89	0.019	0.048	Metabolite up, gene down

Notes: Spearman correlations were calculated between 106 differentially expressed genes ($|\log_2 FC| > 1$, FDR < 0.05) and 9 differentially abundant metabolites (VIP > 1, $p < 0.05$) from the same 6 individual mouse livers. Only pairs with $|\varrho| > 0.6$ and FDR < 0.05 are shown. Bisphenol AF is not registered in HMDB and is indicated as "---". Abbreviations: HMDB, Human Metabolome Database.
